# Research on the impact of live streaming marketing by online influencers on consumer purchasing intentions

**DOI:** 10.3389/fpsyg.2022.1021256

**Published:** 2022-11-11

**Authors:** Xueli Wang, Nadilai Aisihaer, Aihetanmujiang Aihemaiti

**Affiliations:** ^1^Operation Management Department, Gansu Industrial Research Institute of Highway Derivative Economy, Lanzhou, China; ^2^Gansu Province Highway Traffic Construction Group Co., Ltd., Lanzhou, China; ^3^International Business School Suzhou, Xi'an Jiaotong Liverpool University, Suzhou, China; ^4^Human Resources Department, Beijing Changping Technology Innodevelop Group, Beijing, China

**Keywords:** online influencer, purchase intention, consumer behavior-China, live streaming, S-O-R model, e-commerce

## Abstract

Drawing from the stimulus–organism–response (S-O-R) model, this study explores the impact on consumer attitudes in the context of Chinese online influencers' e-commerce live streaming. To examine this impact, we distributed our survey questionnaire to Chinese consumers with live streaming shopping experiences. Using data from 430 valid questionnaires, a hierarchical regression analysis was used to examine our hypotheses. The results show that expertise, bargaining power, post-sales services, and live streaming schedules of online influencers affect consumer trust in online influencers. The expertise, bargaining power, and livestreaming schedules of online influencers affect consumer impulsivity. Moreover, the trust and impulsiveness of online influencers increase consumer purchasing intentions. The implications and future research directions are discussed in this article.

## Introduction

The live streaming economy started in 2019. With the outbreak of COVID-19 in the spring of 2020, a large number of Chinese residents restrained from outdoor activities and started watching live streaming on their mobile phones at home, thus fueling the rapid rise of live streaming platforms such as Tik Tok and Kuaishou. By the late 2020, the number of users watching live streaming in China reached 617 million, accounting for 62.4% of the total number of Internet users (Chong et al., 2018). With the rapid development of e-commerce live streaming, e-commerce live streaming has become the largest category of live streaming, with 388 million users—an increase of 123 million from March 2020 and ~40% of the total number of Internet users. Users who placed orders in the e-commerce live streaming industry accounted for 66.2% of those who watched the live stream, that is, approximately two-thirds of users make purchases after watching e-commerce live streaming (IResearch, 2021). China's e-commerce livestreaming industry is expected to exceed 1.2 trillion yuan annually, with an annual growth rate of 197.0% in the 20th quarter. It is estimated that by 2023, the scale of the e-commerce live streaming industry is expected to exceed 4.9 trillion yuan (IResearch, 2021).

Consumers can clearly and intuitively see the various merchandise information through the interpretation of online influencers in e-commerce livestreams. Online influencers play a role in influencing consumer spending intentions. The role of key opinion leaders (KOL) is the earliest concept proposed by Lazarsfeld et al. (1948) in the People's Choices. The authors think key opinion leaders in interpersonal communication play a positive role, which can provide useful information to others (Park and Lin, 2020). The information recommended by online influencers influences consumer attitudes and buying intentions to a certain extent. Armstrong and Kotler (2012) found that information based on interpersonal interaction is more likely to influence other consumer purchasing desires and trigger consumer purchasing behavior, which is an essential means for enterprises to obtain market value and maintain a competitive advantage. Its role in product diffusion far exceeds that of traditional advertising (Trusov et al., 2009) because consumers compare their own characteristics with online influencer characters, thus easily gaining the trust of consumers. Therefore, this study uses online influencers as an entry point to study the correlated impact of online influencer characteristics on consumer purchasing intentions.

**Figure 1:**
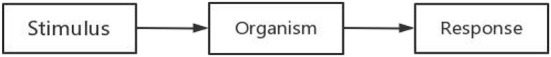
S-O-R model.

The stimulus–organism–response (S-O-R) model (Figure 1), which is a concept derived from psychology, explains the influence of environmental characteristics on user behavior and psychological activities. Based on environmental psychology, Mehrabian and Russell (1974) proposed the S-O-R theoretical model, where S represents stimulus, which has an impact on the subject, and O represents organism. After a certain external environmental stimulus, the corresponding mental activity is generated, and the corresponding behavioral response is defined as R, which can be acceptance or rejection, adoption, or avoidance. The S-O-R model is a general model of human behavior that proposes that consumer purchasing behavior is caused by multiple stimuli. This stimulation comes not only from physiological and psychological factors within the consumers' body but also from external environmental factors. Stimulated by a variety of factors, consumers generate incentives. Motivated by incentives, they make purchasing decisions, implement purchasing behaviors, evaluate the purchased goods and their associated channels and manufacturers after the purchase, and complete the process of making a purchase decision.

Since the S-O-R model has become one of the key theories for studying and explaining user behavior, it has been introduced into different research contexts, including information systems, advertising, e-commerce, and education, covering user behavior, participation behavior, and user comments (Morgan and Hunt, 1994). Belk adopted this model to analyze the influence of environmental variables on consumer behavioral decision-making (Wu and Zhu, 2021). According to the research results, under the stimulation of specific external environmental variables such as shopping scenes and commodity attributes, consumer internal psychological awareness and decision-making will be affected (Li et al., 2022). The S-O-R model has been widely applied in the prediction and interpretation of consumer behavior, such as the research on consumer purchase intention and purchase behavior (Wang and Li, 2017). The theory reveals that product factors such as product price and product category; retailer factors such as promotion strategy, brand, and reputation; and subjective factors such as consumer cognitive emotions and personal experience perception could all influence consumer internal psychological state, which, in turn, affect consumer emotional state and intention to buy (Dennis et al., 2019).

Although some scholars have conducted in-depth research on the impact of consumers' willingness to spend, there is little research on the impact of consumers' willingness to spend and the characteristics of their willingness to buy. Zhao et al. (2020) analyzed the factors that affect consumer consumption, and Falahat et al. (2019) discussed how the online platforms can gain consumer trust and make consumers consume. The innovation of this study comprise the following two aspects: Building on previous studies, this study introduces the influence of online influencers in the context of e-commerce live streaming and discusses the influence of online influencers on consumer spending intentions from a novel perspective and not just in the context of an Internet macro-analysis of how e-commerce platforms drive consumer consumption; the second is the theoretical model of innovation. To our knowledge, this is the first study that considers expertise, post-sales services, bargaining power, and live streaming schedules as independent variables, and trust and impulsiveness as mediating variables to investigate how these variables influence people's purchasing intentions *via* the Internet. In this research, based on the S-O-R model, we consider these four variables as external stimuli: trust and impulse as intermediate variables, consumption intention as the social psychological state of the customer, and consumption intention as the response variable of the customer. According to this theory, a theoretical model under this scenario is creatively proposed to provide optimization recommendations for live streaming marketing.

## Hypothesis development

### Trust as mediator

Since e-commerce cannot take into account face-to-face interactions between buyers and sellers, it is critical to help vendors build a trusting relationship with buyers (Prateek et al., 2016). In previous studies on consumer purchase intentions, online trust was the main barrier to user adoption of e-commerce (Gefen et al., 2003). Therefore, trust is commonly considered by researchers as a highly significant mediating variable. Cheng (2013) also emphasized the importance of establishing a trusted environment with users. Since e-commerce is primarily driven by the subjective belief of consumers that once a deal is made, the other side will keep its word. At the same time, it is also the key to increasing customer loyalty and satisfaction (Dennis et al., 2019). Given the unprecedented power of social media, businesses are increasingly relying on e-commerce as a marketing communication channel (Pauwels et al., 2002). In a mass information society, consumers' willingness to purchase an item can be constrained by uncertain factors. According to the results of research by Reichheld et al. (2000), when consumers become dependent on a particular marketer, they can make purchasing decisions based on trust in a relatively short period of time. Then, Pan et al. (2010) verified that the higher the consumers' trust in the online retailer, the higher the consumers' willingness to purchase the product or service. As pointed out by Cheng (2013), trust is divided into three dimensions: honesty, goodwill, and competence. As the results show, trust has a positive effect on consumer purchasing intentions. In this study, based on the S-O-R model, we use quantitative analysis to verify whether trust as an intermediate variable positively influences consumer purchasing intentions.

Expertise is the degree to which an online influencer can provide relevant knowledge or experience that is correct and valid when consumers perceive information. The attraction of expertise is the power to guide people in a certain direction (Kim and Lennon, 2013). The professionalism of an online influencer refers to the relevant knowledge, experience, or skills that the influencer possesses and disseminates to their followers or other audiences. Rotter (1967) believed that key opinion leaders are people with certain professional knowledge or special charisma who can subtly influence others' attitudes and decisions in a certain way. During the process of watching an online influencer's livestream, the influencer generally gives a lot of professional introductions to their products. The more professional they are, the more consumers perceive that they already know a lot about the product, which reduces the amount of time and cost that consumers need to spend taking the time to understand the product (Tong, 2017). According to Uzunoglu and Kip (2014), fan economy can enhance brand communication between enterprises and consumers. Sokolova and Kefi (2019) study, which analyzed four beauty makeup bloggers, found that physical attractiveness had no significant effect on buying intentions, while credibility and quasi-social interaction increased consumer buying intentions. As a result, professionalism will also lead consumers to trust online influencers more and more, which will enhance consumers' desire to shop.

E-commerce live streaming is different from traditional retail shopping, and there have been cases of items delivered by mail not matching the display. As a result, customers' trust in influencers affects their purchasing intentions. If consumers perceive the post-sales services of live streaming e-commerce as better and more credible, they are relatively more willing to buy.

Following the aforementioned line of research, the following assumptions are made:

***Hypothesis 1:*** *Trust mediates between expertise and purchasing intentions*.
***Hypothesis 2:*** *Trust mediates post-sales services and purchasing intentions*.

Trust is persons' action based on their belief in the character of others. Like any other technological advancement, the Internet has its pros and cons. Influencers can use their public influence and popularity to extract lower prices from product brands, thereby attracting consumers with lower discounts and earning financial benefits. However, according to research, online purchasing systems involve additional risks than traditional purchasing systems, so the vast majority of consumers are price-sensitive (Kacen and Lee, 2002). As a result, consumers look for the best prices and increase their trust in online influencers when they find that an influencer's live streaming is always low or a great deal. Therefore, it proves that trust is closely linked to the cost of the shopping process (Bai et al., 2015). The essence of online influencer monetization is to use a specific channel to convert followers' trust in Internet celebrities into purchasing power in a different way to achieve an economic form of monetization. The fact that consumers enter the live streaming room and watch continuously is evidence that consumers trust a particular influencer (Park and Lin, 2020). Moreover, due to their elevated popularity and following, online influencers have been receiving consistently low prices on the brand side, and these consistently low prices will continue to attract consumer trust in the live streaming rooms of online influencers. Ultimately, trust in influencers drives increased consumer willingness to buy products.

Based on this discussion, hypothesis H3 is proposed:

***Hypothesis 3:*** *Trust mediates bargaining power and purchasing intentions*.

E-commerce live streaming occurs frequently after dinner and into the early hours of the morning. From a scientific point of view, the human brain is excited during the day, where the activity of the rational mind is at its height. During the night, activity in the sensory systems of the brain increases, as does emotional activity (Xie et al., 2019). According to consumer psychology, consumers are irrational in their decision-making process and are commonly influenced by various environmental factors (Butt et al., 2022). In particular, when their favorite online influencers stream live, consumers make faster purchase decisions due to their trust in them.

Therefore, hypothesis H4 is proposed:

***Hypothesis 4:*** *Trust mediates the live streaming schedule and purchasing intentions*.

### Impulsiveness as mediator

Impulsive consumption is the unplanned or conscious purchasing behavior of customers that is driven by external factors. According to Piron's definition, stimulation can also come from the goods, the shopping environment, and other people (Zheng et al., 2019). Furthermore, the characteristics of online influencers will also be an essential stimulus for consumers to make impulse purchases. In e-commerce live streaming, consumer attention has shifted primarily from products to online influencers (Ma and Mei, 2018). The more professional the influencers are, the better the delivery efficiency and effect of product information will be and the more the consumers think they can get more detailed information about products from online influencers (Meng et al., 2020). At this point, consumers will assume that the expertise of online influencers has guided their purchases and will therefore make impulse purchases.

Based on the these research ideas, we propose the following hypothesis:

***Hypothesis 5:*** *Impulsiveness mediates between expertise and purchasing intentions*.

Promotion is an effective marketing tool to influence consumer shopping decisions. Large-scale promotions generally lead to quick decisions and impulse purchases by consumers (Stanko, 2016). The higher the price discount, the higher the consumers' shopping satisfaction and the stronger the sense of purchase, which may lead the consumers to buy non-essential items or make additional purchases (Yu et al., 2018). The result is that the more favorable product prices are, the more consumers are willing to pay, even for discretionary items.

Therefore, we propose hypothesis H6:

***Hypothesis 6:*** *Impulsiveness mediates bargaining power and purchasing intentions*.

The choice of time period for live streaming is significant. Platform users have their own usage habits, but most consumers use fragmented time to watch videos so as to make up for their dull time (Ba and Paul, 2002). According to research, people's desire to buy continues to increase after returning home from getting off work at night in China (Liao et al., 2020). Companies and users who choose to launch live streaming during these periods can then capture the attention of legions of consumers, gain fans, and viewers and thus stimulate consumer willingness to spend. Hence, we propose the hypothesis:

***Hypothesis 7:*** *Impulsiveness mediates live streaming schedules and purchasing intentions*.

### Purchasing intentions

Consumer purchasing behavior is one of the most relevant research topics in the existing research community and has strong research value. Fishbein (1967) defines purchase intention as the subjective preference of consumers to purchase products or services. In this study, purchase intent refers to the behavioral likelihood of a consumer to purchase a product or service during an e-commerce live streaming event. According to the practice of Zhang and Benyoucef (2016), the factors affecting consumer purchase intention can be divided into two categories based on the S-O-R model: one is the objective stimulus representing the stimulus, and the other is the customer perception factor representing the organism (Koo and Ju, 2010), which can be described in terms of impulsiveness and trust. Therefore, the study of trust and impulsiveness had certain enlightening significance for the implementation of improvement measures of consumer purchase intention in e-commerce live streaming (Zhao et al., 2019). Thus, we suggest the following assumptions:

***Hypothesis 8:*** *Trust has a positive effect on purchasing intentions*.
***Hypothesis 9:*** *Impulsiveness has a positive effect on purchasing intentions*.

**Figure 2:**
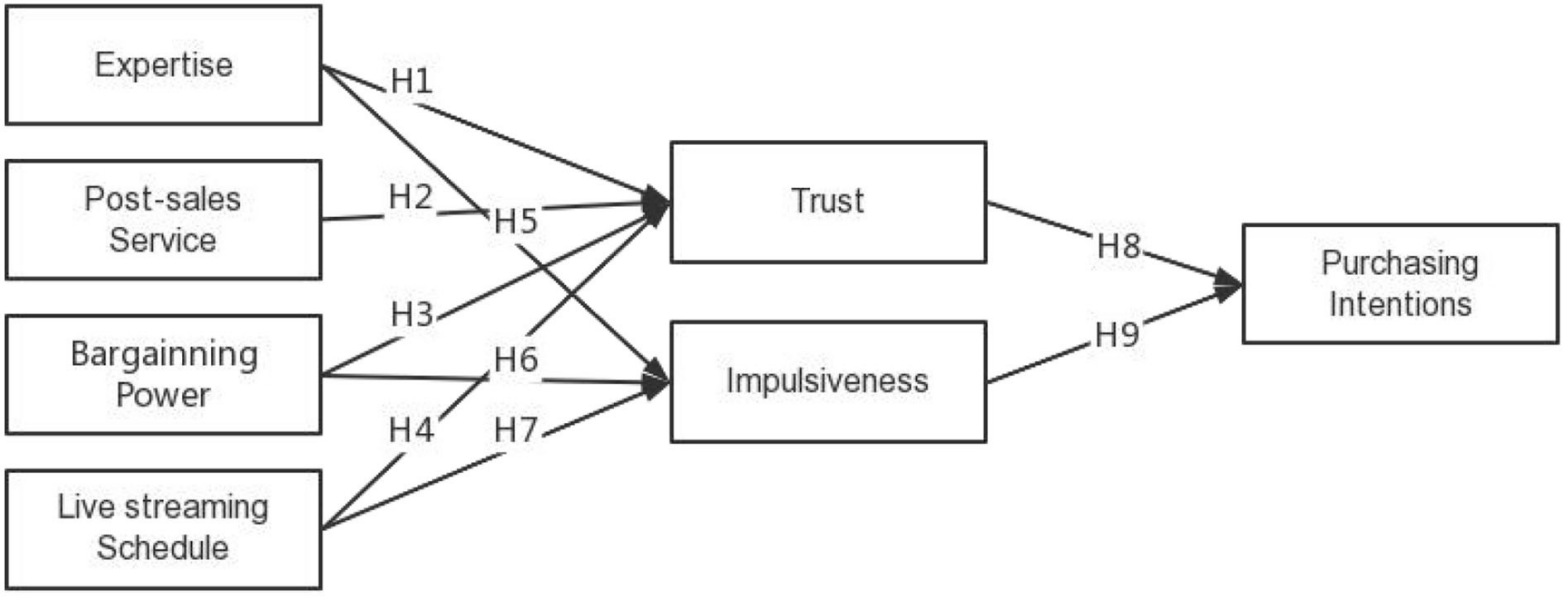
Theoretical model in this study.

The assumptions presented in the present study are summarized in Figure 2.

## Materials and methods

### Participants and procedures

To test our hypothesis, we collected data using an online survey on a social network platform and a snowball sampling method for human relations. The three writers are frequent buyers on live streaming platforms and are familiar with the personalities of various online influencers. Following a snowball sampling procedure, the three authors distributed survey links to participants and rapidly disseminated them in a human-to-human fashion. This approach has been widely used and is effective for collecting data (Yang et al., 2021). Second, we also uploaded questionnaire links on social media sites to attract additional participants. Each participant was informed that their information in this survey will be anonymous and confidential, and that all data collected will be used for research purposes only. The questionnaire assessed participants' recognition of characteristics such as influencer professionalism, post-sales service, live streaming time, and bargaining power, which ultimately influence consumer purchasing intentions.

After repeated investigation and final review, a total of 520 questionnaires were obtained, of which 430 were valid, with a response rate of 82.7%. There were 206 male respondents, or 47.9%, and 224 female respondents, or 52.1%, with an average age of 32.74 years. In terms of income, 29.8% of the respondents earned <5,000 yuan, 46.6% earned between 5,000 and 10,000 yuan, and 23.6% earned more than 10,000 yuan. In addition, the participants had different levels of education. Respondents with a 3-year college degree or less accounted for 27.3%, those with a bachelor's degree accounted for 58.9%, and those with a master's degree or above accounted for 13.8%.

### Measures

A 5-point Likert scale was used to measure all variables in the questionnaire (with 1 = firmly disagree and 5 = fully agree) in this survey. Some of questions of trust are designed according to the article by McAllister (1995). Zhou et al. (2020) used cognitive trust and affective trust classification to study the trust relationship between consumers; thus, related scales were used in this questionnaire survey. Fu (2020) mainly studied the influence of KOL on consumer purchase intentions, given that KOL and online influencers are extremely similar in some areas, especially in terms of promotion and impact on consumers. This scale is used as a reference throughout this article. This study also refers to the scale from Tsalis (2020) to study the impact of price on purchase intention. To ensure accurate translation of various scales, each item of each construct followed the reverse translation procedure suggested by Brodie et al. (2013).

#### Reliability and validity tests

This study uses the Statistical Package for the Social Science Software Automatically (The SPSSAU Project, 2022) to analyze the reliability of the questionnaire and analyzes the internal reliability of online influencers' expertise, post-sales service, bargaining power, live streaming schedule, trust, impulsiveness, and purchase intentions. First, we analyze the coefficients in this study. If the coefficient is >0.8, the reliability is high (Eisinga et al., 2013). Table 1 shows that the Cronbach coefficient for each scale is above 0.8, indicating the strong reliability of the scales.

**Table 1:** Results of reliability and validity tests.

**Variables**	**Cronbach reliability analysis**		**Validity analysis results**			
			**Coefficient**			**Common factor variance**
	**Items**	**Cronbach α**	**Factor 1**	**Factor 2**	**Factor 3**	
Expertise	X11: Online influencers' explanation and introduction can let me quickly understand the product	0.929	0.677	0.335	0.438	0.762
	X12: I consider him to be an expert in this field		0.8	0.253	0.323	0.808
	X13: I think he has enough experience to make a judgement on this kind of product		0.812	0.2	0.347	0.82
Post-sales service	X21: It is very important to me whether there is no reason to return or exchange for seven days	0.933	0.763	0.314	0.257	0.747
	X32: It means a lot to me whether I can return it for free		0.318	0.179	0.735	0.674
	X33: Delivery speed after purchase is very important to me		0.299	0.234	0.76	0.722
Bargaining power	X41: He always spends a lot of time looking for the best deal	0.887	0.234	0.273	0.819	0.8
	X42: Because I know he has the best price so I always purchase in his studio		0.154	0.285	0.813	0.766
	X43: His promotion is valuable and special		0.268	0.238	0.825	0.809
Live streaming schedule	X51: I purchase more in the evening than I do in the daytime	0.794	0.398	0.355	0.673	0.737
	X52: The live streaming time is always when I am free and relaxed, usually in the evening		0.601	0.436	0.334	0.664
	X53: When I come to my senses during the day, I regret the impulse purchases I made during the night		0.583	0.438	0.458	0.741
Trust	Y11: I believe there will be no problem with the product he recommended	0.937	0.452	0.487	0.396	0.598
	Y12: I can rely on the information he gives to make a sufficient judgment on the product		0.469	0.521	0.388	0.642
	Y13: I think the information he provided corresponds to the actual situation of the goods		0.196	0.601	0.303	0.491
Impulsiveness	Y21: At night, my emotions are easily emotional and impulsive, which can be easily induced by live streaming	0.907	0.685	0.525	0.134	0.762
	Y22: I always buy things on impulse because I watch them on live streaming		0.707	0.497	0.269	0.819
	Y23: I can't help buying when I see a good deal		0.674	0.506	0.297	0.798
Purchasing intention	Y31: I think the product he recommended is worth buying	0.940	0.347	0.706	0.343	0.737
	Y32: I want to try the product he recommended		0.306	0.829	0.258	0.848
	Y33: I will recommend the products he recommended to my family and friends		0.223	0.79	0.315	0.773
	Y34: He changed my mind about the product		0.53	0.709	0.272	0.858
Characteristic root value (before rotation)			15.741	1.836	1.18	–
Variance interpretation rate % (before rotation)			62.965%	7.343%	4.721%	–
Cumulative variance interpretation rate % (before rotation)			62.965%	70.308%	75.029%	-
Characteristic root value (after rotation)			6.653	6.603	5.502	–
Variance interpretation rate % (after rotation)			26.611%	26.411%	22.007%	–
Cumulative variance interpretation rate % (after rotation)			26.611%	53.022%	75.029%	–
KMO			0.961	–		
Bartlett spherical value			10,545.763	–		
*df*			300	–		
*p*			0	–		

In terms of structural validity, the data analysis method of factor analysis is used in this study. The KMO values, covariance explanatory rate values, factor loading coefficient values, and additional metrics are used separately in the combined analysis to validate the level of validity of the data. The KMO value is used to judge validity, common degree value is used to exclude unreasonable research projects, variance interpretation rate value is used to describe the information extraction level, and factor loading coefficient is used to measure the corresponding relationship between factors (dimensions) and projects. As can be seen from Table 1, all research items have a common degree value >0.4, indicating that the information of research items can be efficiently extracted. In addition, the variance interpretation rate values of the three factors are 26.611, 26.411, and 22.007, respectively, and the cumulative variance interpretation rate after rotation is 75.029% > 50%, indicating that the information of the research project can be efficiently extracted. Second, the KMO and Bartlett tests are used to verify the validity. As can be seen from Table 2, the KMO value is 0.961, which is >0.8, meeting the requirements (Chung et al., 2004). The Bartlett test is used for validity analysis validity analysis, which has a *P* value of 0, showing the validity of the studied data is excellent.

**Table 2:** Results of mediation analysis (*n* = 430).

	**Purchasing intentions**					**Impulsiveness**					**Trust**					**Purchasing intentions**				
	**B**	**Standard error**	**t**	***p***	**β**	**B**	**Standard error**	**t**	***p***	**β**	**B**	**Standard error**	**t**	***p***	**β**	**B**	**Standard error**	**t**	***p***	**β**
Constant	−0.121	0.216	−0.562	0.574	–	0.211	0.241	0.876	0.382	–	0.129	0.200	0.644	0.520	–	−0.262	0.159	−1.646	0.101	–
Live streaming schedule	0.387^**^	0.049	7.959	0.000	0.374	0.531^**^	0.054	9.757	0.000	0.503	0.264^**^	0.045	5.855	0.000	0.264	0.050	0.041	1.233	0.218	0.048
Bargaining power	0.267^**^	0.055	4.829	0.000	0.275	0.166^**^	0.062	2.678	0.008	0.167	0.313^**^	0.051	6.105	0.000	0.333	0.096^*^	0.043	2.241	0.026	0.099
Post-sales service	0.003	0.037	0.082	0.935	0.003	0.057	0.041	1.392	0.165	0.062	−0.078^*^	0.034	−2.310	0.021	−0.091	−0.003	0.027	−0.098	0.922	−0.003
Expertise	0.269^**^	0.045	5.997	0.000	0.270	0.154^**^	0.050	3.078	0.002	0.152	0.393^**^	0.042	9.446	0.000	0.408	0.080^*^	0.037	2.187	0.029	0.081
Impulsiveness																0.493^**^	0.036	13.750	0.000	0.503
Purchasing intentions																0.285^**^	0.043	6.613	0.000	0.277
*R^2^*	*0.709*					*0.651*					*0.733*					*0.843*				
*Adjust R^2^*	*0.706*					*0.647*					*0.730*					*0.840*				
*F value*	*F _(4,370)_ = 225.702, p = 0.000*					*F _(4,370)_ = 172.288, p = 0.000*					*F _(4,370)_ = 254.058, p = 0.000*					*F _(6,368)_ = 328.838, p = 0.000*				

* p < 0.05;

** p < 0.01.

## Data analysis and result

### Hypothesis testing

#### Stratification regression analysis

Regression analysis mainly determines the causal relationship between variables. In this analysis, the expertise, live streaming schedule, bargaining power, and post-sales services of online influencers are considered independent variables X; trust and impulsiveness as intermediary variables M; and purchase intentions as dependent variables Y. The influence relationship between them is analyzed by hierarchical regression. The reason for using hierarchical regression in this study is that the theoretical model includes some mediator variables. The study by Chen et al. (2019) showed that hierarchical regression is suitable for models with mediating variables; hence, we use this for analysis. The results are shown in Table 2.

It can be seen from Table 2 that the mediation effect analysis involves three models, which are given as follows:

Purchase intentions = −0.121 + 0.387 ^*^ Live streaming schedule + 0.267 ^*^ Bargaining power + 0.003 ^*^ Post-sales service + 0.269 ^*^ Expertise.
Impulsiveness = 0.211 + 0.531 ^*^ Live streaming schedule + 0.166 ^*^ Bargaining power + 0.057^*^ Post-sales service.
Trust = 0.129 + 0.264 ^*^ Live streaming schedule + 0.313 ^*^ Bargaining power – 0.078 ^*ast*^ Post-sales service + 0.393 ^*^ expertise.
Purchase intentions = −0.262 + 0.050 ^*^ Live streaming schedule + 0.096^*^ Bargaining Power – 0.003^*^ Post-sales service + 0.080^*^ Expertise + 0.493 ^*^ Impulsiveness + 0.285 ^*^ Trust.

#### Mediating effect test

There are six relevant indicators involved in the study of mediation effects, which are described in Table 3.

**Table 3:** Summary of mediating effect test results.

**Item**	**c**	**a**	**b**	a^*^b	a^*^b	a^*^b	a^*^b	a^*^b	**c' direct**	**Test**
	**Total effect**			**The mediation effect**	**(boot SE)**	**(*z value*)**	**(*p value*)**	**(95% BootCI)**	**effect**	**conclusion**
LS→ IM→ PI	0.387^**^	0.531^**^	0.493^**^	0.261	0.043	6.048	0.000	0.173 to 0.344	0.050	Complete mediation
LS→ T→ PI	0.387^**^	0.264^**^	0.285^**^	0.075	0.024	3.159	0.002	0.034 to 0.126	0.050	Complete mediation
BP→ IM→ PI	0.267^**^	0.166^**^	0.493^**^	0.082	0.045	1.829	0.067	0.003 to 0.180	0.096^*^	Partial mediation
BP→ T→ PI	0.267^**^	0.313^**^	0.285^**^	0.089	0.027	3.356	0.001	0.046 to 0.148	0.096^*^	Partial mediation
PS→ IM→ PI	0.003	0.057	0.493^**^	0.028	0.033	0.851	0.395	−0.028 to 0.100	−0.003	Not significant
PS→ T→ PI	0.003	−0.078^*^	0.285^**^	−0.022	0.014	−1.587	0.112	−0.055 to 0.001	−0.003	Complete mediation
E→ IM→ PI	0.269^**^	0.154^**^	0.493^**^	0.076	0.032	2.404	0.016	0.016 to 0.140	0.080^*^	Partial mediation
E→ T→ PI	0.269^**^	0.393^**^	0.285^**^	0.112	0.027	4.230	0.000	0.064 to 0.169	0.080^*^	Partial mediation

* p < 0.05;

** p < 0.01.

LS, live streaming schedule; IM, impulsiveness; PI, purchasing intentions; T, trust; E, expertise; BP, bargaining power; PS, post-sales service.

A is the regression coefficient of X on M; B is the regression coefficient of M on Y; A ^*^ B is the product of A and B, that is, the mediating effect; and 95% BootCI indicates the 95% confidence interval calculated using bootstrap sampling. It is significant if the interval does not contain 0. If both A and B are significant and C′ is trivial, then this is a complete concord. If A and B are significant, C′ is significant, and A ^*^ B is of the same sign as C′, it is a partial mediating effect. If at least one of A and B is not significant and the 95% BootCI of A ^*^ B includes the number 0 (not significant), the mediating effect is not significant. If at least one of A and B is not significant, the 95% BootCI of A ^*^ B does not include the number 0 (significant), and C′ is not significant, it is fully mediated. If at least one of A and B is not significant, the 95% BootCI of A ^*^ B does not include the number 0 (significant), C′ is significant, and A ^*^ B and C′ have the same sign, then it is a partial mediation effect.

#### Hypothesis testing results

To further test our hypotheses, we performed a series of hierarchical multivariate regression analyses. The final results are shown in Table 4. In the hypothesis model, trust acts as a mediator in terms of expertise, post-sales services, bargaining power, live streaming schedules, and purchase intentions. Moreover, expertise, post-sales services, bargaining power, live streaming schedule, and purchasing intentions are positively correlated. Thus, assumptions 1, 2, 3, 4, and 8 are supported.

**Table 4:** Summary of mediation effect size results.

**Item**	**Inspection conclusion**	**c** **Total effect**	a*b **The mediation effect**	**c′** **Direct effect**	**Calculation formula of effect proportion**	**% of effect**
Live streaming schedule→ Impulsiveness→ Purchasing intentions	Complete mediation	0.387	0.261	0.050	–	100%
Live streaming schedule→ Trust→ Purchasing intentions	Complete mediation	0.387	0.075	0.050	–	100%
Bargaining power→ Impulsiveness→ Purchasing intentions	Partial mediation	0.267	0.082	0.096	a*b/c	30.561%
Bargaining power→ Trust→ Purchasing intentions	Partial mediation	0.267	0.089	0.096	a*b/c	33.504%
Post-sales service→ Impulsiveness→ Purchasing intentions	Was not significant	0.003	0.028	−0.003	–	0%
Post-sales service→ Trust→ Purchasing intentions	Complete mediation	0.003	−0.022	−0.003	–	100%
Expertise→ Impulsiveness→ Purchasing intentions	Partial mediation	0.269	0.076	0.080	a*b/c	28.289%
Expertise→ Trust→ Purchasing intentions	Partial mediation	0.269	0.112	0.080	a*b/c	41.746%

The validation results show that impulsivity has a moderating effect on professionalism, bargaining power, live streaming schedule, and purchasing intentions, and professionalism, bargaining power, live streaming time, and purchasing intentions are positively correlated. Thus, assumptions 5, 6, 7, and 9 are supported.

## Discussion

### Theoretical contributions

There are three theoretical contributions to our study. First, by reviewing relevant literature on influencing factors of consumer behavior on live streaming platforms, this study contributes to the literature on e-commerce (Wongkitrungrueng and Assarut, 2020). With the popularity of live streaming, consumers have more and more choices for self-entertainment, and the online influence economy is also growing (Hollebeek et al., 2014). In addition, our study adds to the growing body of research on how influencers' bargaining power and live streaming schedules influence consumer purchasing intentions. Although studies show that post-sales service has a significant impact on consumer purchase intention, the impact on consumer impulse consumption is not obvious enough (Bai et al., 2015). This study introduced consumer trust and impulsiveness in the theoretical model (Wu and Zhu, 2021) according to the concepts derived from S-O-R model research (Mehrabian and Russell, 1974). From the point of view of purchase intent, our results suggest that the relationship between consumer trust in online influencers and consumer impulse spending acts as mediator. On the one hand, the mediator model enriches the action scenario of the model. On the other hand, it helps businesses and users to better understand consumer spending intentions.

## Conclusion

In this study, the S-O-R model and two mediating variables, trust and impulsiveness, are used to further explore the relationship between the professionalism, bargaining power, post-sales services, and live streaming schedules of online influencers, and consumer purchasing intentions. The results show: first, professionalism, bargaining power, post-sales services, and live streaming schedules of online influencers can enhance consumer trust in them. The higher the consumers' trust in an online influencer, the more favorable it will be for improving the consumers' purchasing intentions. In other words, trust acts as an intermediary between professionalism, bargaining power, post-sales services, and live streaming schedules of online influencers, and consumer purchasing intentions. Second, professionalism, bargaining power, and live streaming schedules of online influencers can enhance the impulsive nature of consumer purchasing intentions. The more impulsive consumers are when watching live streaming shopping, the better it is for boosting consumer buying intentions. As a result, impulsivity mediates the relationship between the professionalism, bargaining power, and live streaming schedules of online influencers and consumer buying intentions.

### Practical implications

From a business and brand perspective, live streaming provides a better sales channel than traditional retail and online shopping. The most popular online influencers such as Li Jiaqi and Luo Yonghao have created sales records: Li's sales reached 12.13 billion yuan on 20 October 2021 (Bai et al., 2015), while Luo Yonghao's goods sales on his first Tik Tok live streaming reached more than 100 million yuan (Center for Social Media Research School of New Media Peking University, 2020). Live streaming with well-known online influencers can be a better sales channel for enterprises and brands to enter the market.

From the point of view of online influencers, the four characteristics discussed in this study can be used to increase the purchasing intentions of consumers and hence the turnover of live streams. The most famous example is Jiaqi Li, who is called “Lipstick Brother No. 1” on the Internet. He used to work in cosmetics sales, and his knowledge of cosmetics is highly professional. His fan base is made up of beauty-conscious young women, and he mainly recommends women's cosmetics. Because of his expertise, female cosmetics consumers trust him so much that they are willing to buy cosmetics from his studio. At the same time, because of its huge sales, for instance, his sales reached 12.13 billion yuan on 20 October 2021, brands always offer him the lowest price in the whole Internet so as to attract more consumers and form a virtuous circle (Brodie et al., 2013).

From a consumer perspective, the professionalism of influencers, their bargaining power, post-sales services, and live streaming schedules discussed in this study are all criteria for consumers to measure influencers, thus continuously raising the threshold for influencers to enter the industry. As a result of the market competition in this industry, a large number of influencers have been gradually eliminated by the market, which proves that the market competition is constantly improving the professional quality of influencers and standardizing e-commerce live streaming, so as to better meet the rights and interests of consumers. Influencers need to accurately target their audience and schedule their live content accordingly. It is also necessary to continuously improve product quality and enhance customer trust to achieve healthy product sales development.

The government argues that live streaming appears to lack regulation, such as Viya's tax evasion. In addition to tax evasion, false claims, data fraud, the sale of counterfeit goods, poor post-sales services, and the additional problems that keep popping up, the industry as a whole has uncovered a large number of normative loopholes during its massive growth. The government is continuously optimizing the environment for e-commerce live streaming. Consumers should understand ways to safeguard their rights and interests. The code of conduct for e-commerce live streaming was published for the first time in 2020 in China, clarifying the duties, obligations, and rights of merchants, influencers, platforms, and other participants in the e-commerce live streaming industry. Consumers can understand the relevant content and enhance their risk awareness. Consumers can use laws to protect their rights and interests, such as the Consumer Rights and Interests Protection Act of the People's Republic of China, to safeguard their legitimate rights and interests and protect themselves.

## Limitations and future research directions

Although our study has yielded certain results, there are some gaps. First, in terms of the study sample, although the questionnaire was publicly available on the Internet, the non-probabilistic sampling resulted in a regionally selective bias in the study sample. Most of the samples were from a limited number of Chinese provinces, while fewer samples were from different provinces. The convenience of having samples from the same region and in the same company allowed educational background and income and other conditions to deviate from reality. There is a large gap between the two sets of data in the analysis of the multi-group structure equation model. It is expected that future studies will continue to improve the collection of sample data. Second, the choice of variables is not comprehensive. The environmental stimulus variables involved in e-commerce live streaming are limited, and other forms of stimulus, such as text, are not included. Third, the recall method used in data collection does not guarantee data recovery quality. Therefore, in future, relatively immediate feedback methods such as neurophysiology can be used to identify research variables and improve the precision of variable measurements.

## Data availability statement

The original contributions presented in the study are included in the article/supplementary material, further inquiries can be directed to the corresponding authors.

## Ethics statement

Ethical review and approval was not required for the study on human participants in accordance with the local legislation and institutional requirements. Written informed consent from the patients/participants or patients/participants legal guardian/next of kin was not required to participate in this study in accordance with the national legislation and the institutional requirements.

## Author contributions

XW developed the theoretical framework and worked on literature review, data collection and analysis, and manuscript writing. NA contributed to the literature review, theoretical implications, and manuscript writing. AA worked on the manuscript. All authors contributed to this article and approved the submitted version.

## Conflict of interest

Author XW was employed by Gansu Province Highway Traffic Construction Group Co., Ltd. Author AA was employed by Beijing Changping Technology Innodevelop Group. The remaining author declares that the research was conducted in the absence of any commercial or financial relationships that could be construed as a potential conflict of interest.

## Publisher's note

All claims expressed in this article are solely those of the authors and do not necessarily represent those of their affiliated organizations, or those of the publisher, the editors and the reviewers. Any product that may be evaluated in this article, or claim that may be made by its manufacturer, is not guaranteed or endorsed by the publisher.
